# Delayed methicillin-resistant *Staphylococcus aureus*-induced osteomyelitis of the tibia after pin tract infection: two case reports

**DOI:** 10.1186/s13256-016-1187-x

**Published:** 2017-01-31

**Authors:** Kosuke Hamahashi, Yoshiyasu Uchiyama, Yuka Kobayashi, Masahiko Watanabe

**Affiliations:** Department of Orthopaedic Surgery, Tokai University School of Medicine, 143 Shimokasuya, Isehara, Kanagawa 259-1193 Japan

**Keywords:** Case report, External fixation, Methicillin-resistant *Staphylococcus aureus*, Osteomyelitis, Pin tract infection

## Abstract

**Background:**

Pin tract infection is a common complication of external fixation. It usually heals after treatment with debridement, antibiotics, and/or pin removal, only rarely developing into delayed osteomyelitis. We treated two patients with delayed methicillin-resistant *Staphylococcus aureus*-induced osteomyelitis of the tibia following pin tract infection.

**Case presentation:**

One patient, a diabetic 60-year-old Japanese man, underwent definitive external fixation using an Ilizarov fixator for postoperative osteomyelitis of an open fracture of his left ankle. One year after removing the external fixator, he developed methicillin-resistant *Staphylococcus aureus*-induced osteomyelitis of the tibial pin site. He underwent surgical debridement four times. No recurrence was seen 2 years 8 months after the last debridement. Another patient, a healthy 38-year-old Japanese man, underwent bilateral temporary external fixation for a right ankle open fracture and a comminuted fracture of the left tibial plateau. Three months after removal of the external fixator, he was diagnosed with methicillin-resistant *Staphylococcus aureus*-induced osteomyelitis of the bilateral tibial pin sites. He underwent surgical debridement three times, but the infection of his right tibia persisted. Finally, a gastrocnemius muscle flap was placed. No recurrence was seen 2 years after this last surgery.

**Conclusions:**

Pin tract infection should not be considered a minor complication because osteomyelitis may develop, requiring treatment that is more aggressive than curettage of the pin tract. A gastrocnemius flap is a useful treatment option for refractory osteomyelitis because flap harvest causes less functional disturbance and is a relatively easy surgical technique.

## Background

Temporary external fixation is applied for a limited time to treat open fractures and periarticular fractures associated with severe soft tissue damage. It is also recommended for damage control orthopedics in patients with multiple injuries [1]. Definitive ring fixation is applied for longer periods to address infected nonunion or large bone defects [2]. Pin tract infection (PTI) is a common complication associated with both temporary and definitive external fixation [3]. It usually heals after treatment with debridement, antibiotics, and/or pin removal, only rarely developing into delayed osteomyelitis. Surgical wounds are most at risk of methicillin-resistant *Staphylococcus aureus* (MRSA) infection. MRSA infections are worse than those caused by other pathogens because of the limited choice of available efficacious antibiotics, thus making it difficult to eradicate these strains. The MRSA isolation rate in healthcare settings is higher in Japan than in other countries [4, 5]. We report the treatment of two rare cases of delayed MRSA-induced osteomyelitis of the tibia after PTI.

## Case presentation

### Case 1

**Fig. 1:**
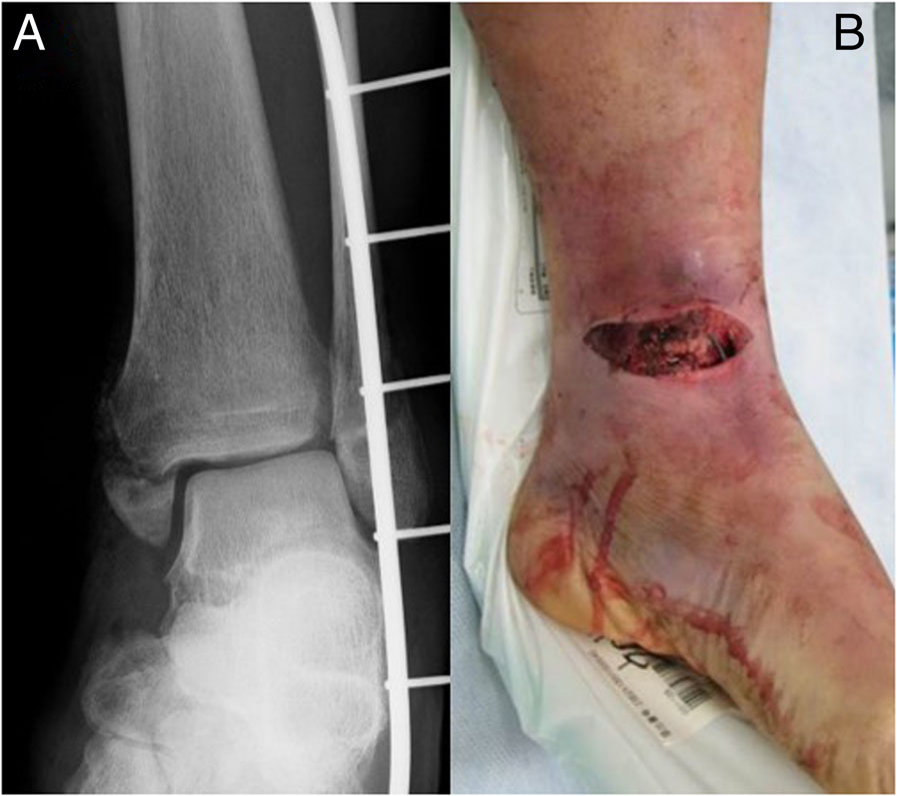
Anteroposterior plain radiography of the *left ankle* (**a**) and physical appearance of the open wound (**b**). The injury was classified as a Gustilo–Anderson 3B, AO classification 44-B2 open fracture

**Fig. 2:**
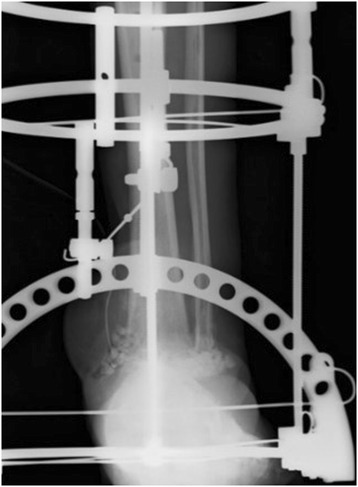
Anteroposterior plain radiography of the *left ankle*. An Ilizarov external fixator was applied after removing the internal fixation implant because of a deep infection

**Fig. 3:**
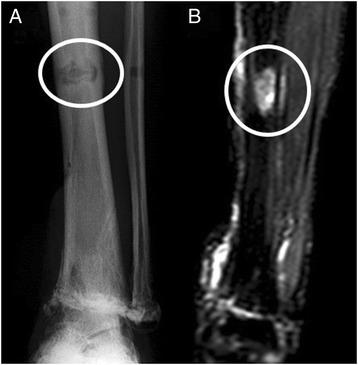
Anteroposterior view of an osteolytic lesion. Plain radiography shows an osteolytic lesion in the tibial pin tract (**a**). T2-weighted magnetic resonance imaging shows fluid collection (**b**). The circle shows osteolytic lesion (**a**), and fluid collection (**b**), respectively

**Fig. 4:**
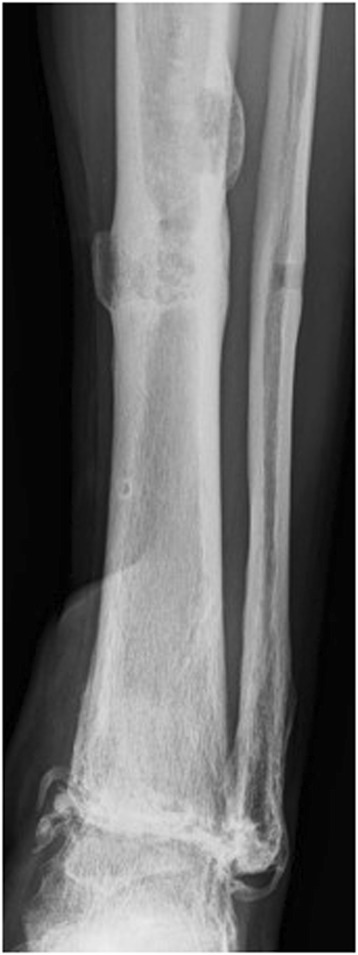
Anteroposterior radiography of the *left ankle*. There is no recurrence of osteomyelitis 2 years 8 months postoperatively

The patient was a diabetic 60-year-old Japanese man who experienced an open fracture of his left ankle (Gustilo–Anderson classification 3B, AO classification 44-B2; Fig. 1). He underwent internal fixation complemented by an epigastric artery perforator flap. He had poorly controlled diabetes, as shown by a blood glucose concentration of 361 mg/dl and glycated hemoglobin (HbA1c) level of 8.2 %. Six months after the surgery, the fracture site developed a deep infection. The wound culture was positive for MRSA. The internal fixation implant was removed, and an Ilizarov external fixator was applied. Arthrodesis was performed with several fine wires inserted into his tibia and metatarsal bones (Fig. 2). The Ilizarov external fixator was removed after the infection had healed and arthrodesis was complete. The external fixation period was 6 months. During this period, once-daily showering and pin site care with chlorhexidine solution 2 mg/ml was administered by the patient himself. One year later, discharge of pus from one of the tibial pin tracts was detected, and MRSA was diagnosed by bacteriological tests. Plain radiography showed an osteolytic lesion at the tibial pin tract. T2-weighted magnetic resonance imaging showed fluid collection in his bone marrow (Fig. 3). The diagnosis was osteomyelitis. He underwent surgical debridement four times. An autogenous bone graft was finally placed. Beginning at the time of the first debridement, vancomycin was administered intravenously for 10 weeks (1.5 to 2.5 g/day, depending on the monitored blood level), followed by oral rifampicin and trimethoprim-sulfamethoxazole for 7 months. No recurrence was seen 2 years 8 months after the last surgery (Fig. 4).

### Case 2

**Fig. 5:**
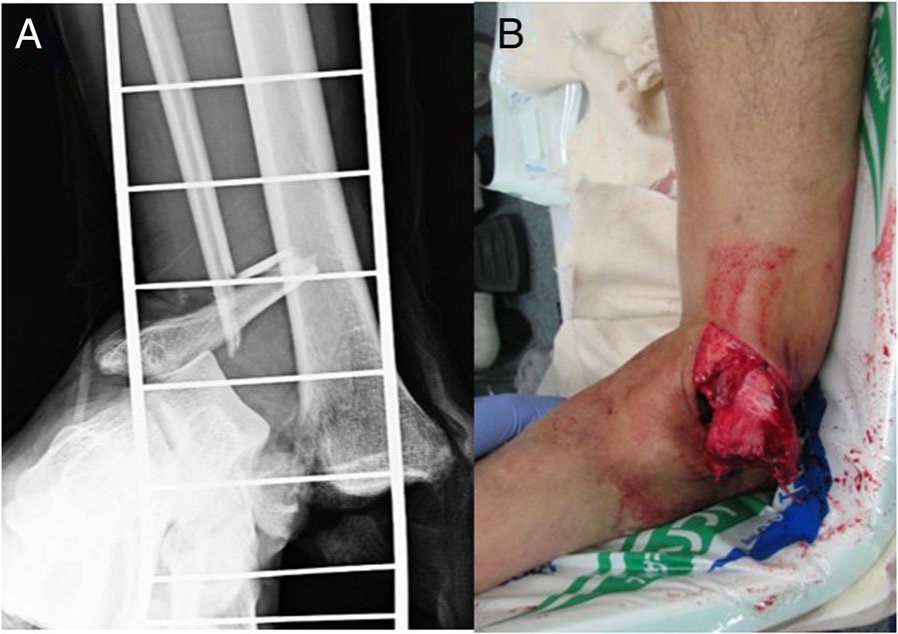
Anteroposterior radiography (**a**) and physical appearance (**b**) of the *right ankle*. It was classified as a Gustilo–Anderson 3A, AO classification 44-C2 open fracture

**Fig. 6:**
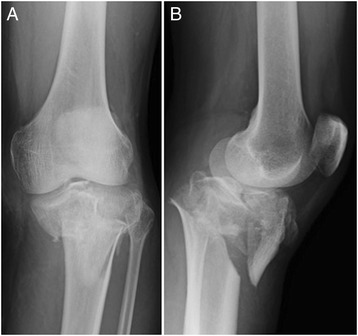
Anteroposterior (**a**) and lateral (**b**) radiography of the *left knee*. It was classified as an AO 41-C2 comminuted fracture of the tibial plateau

**Fig. 7:**
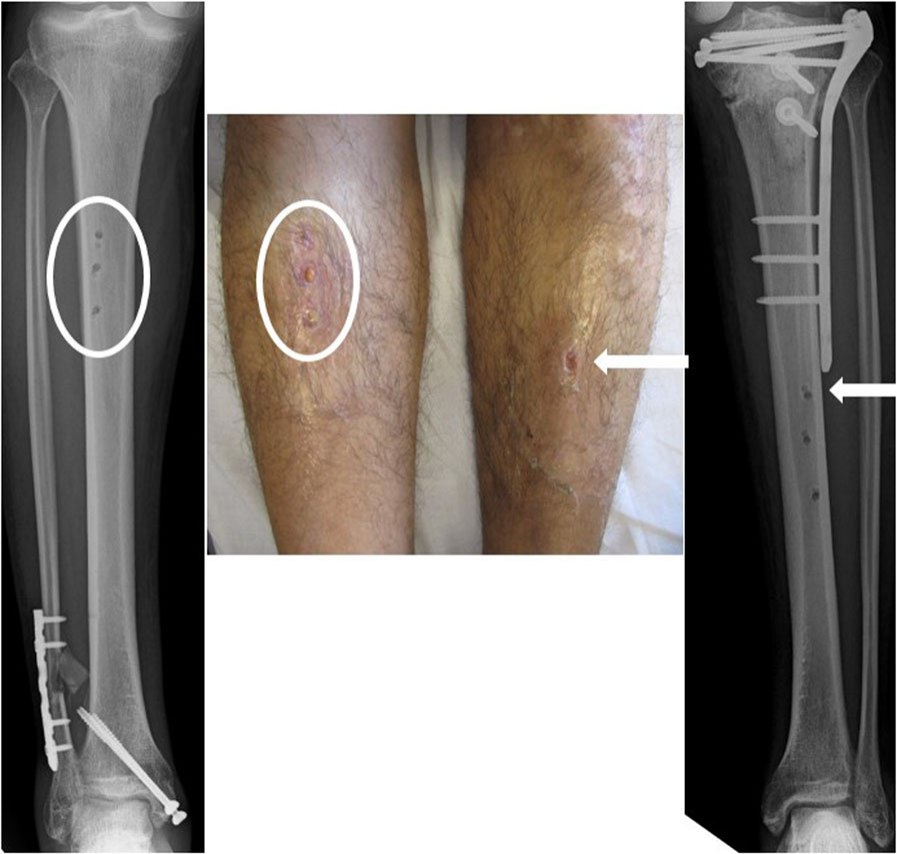
Anteroposterior radiography and physical appearance of both *lower legs*. There was discharge of pus from the bilateral tibial pin tracts (*encircled in white* on the *right tibia* and shown by *white arrows* on the *left tibia*)

**Fig. 8:**
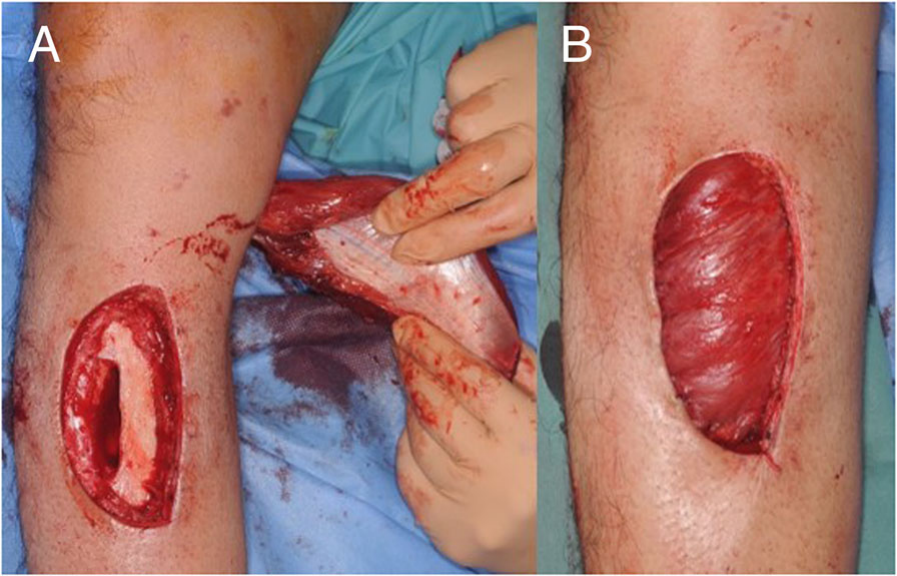
Intraoperative view of the *right lower leg*. The gastrocnemius muscle flap was harvested (**a**) and transplanted into the medullary cavity through 10×30 mm cortical bone fenestration (**b**)

**Fig. 9:**
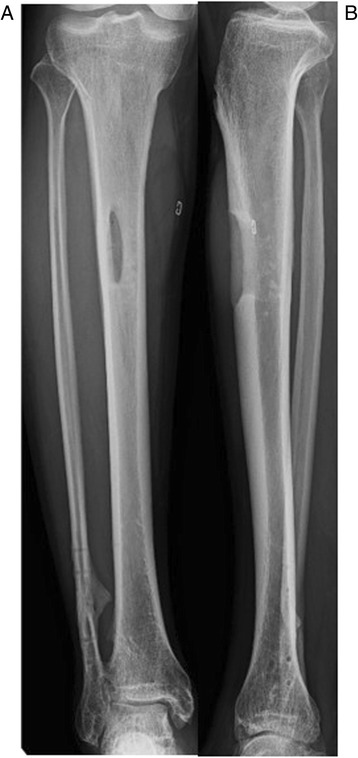
Anteroposterior (**a**) and lateral (**b**) radiography of the *right lower leg*. There is no recurrence of osteomyelitis 2 years postoperatively

The patient was a healthy 38-year-old Japanese man who experienced an open fracture (Gustilo–Anderson classification 3A, AO classification 44-C2) of his right ankle (Fig. 5) and a comminuted fracture (AO classification 41-C2) of his left tibial plateau (Fig. 6). He underwent bilateral temporary external fixation with three half-pins inserted into his tibia, two penetrating pins inserted into the calcaneus for his ankle fracture and with three half-pins inserted into his tibia and femur for the tibial plateau fracture. They were later converted to internal fixation. The external fixation period was 18 days. During this period, the surgeons or nursing staff provided once-daily pin site care with chlorhexidine solution 2 mg/ml. Three months after removing the external fixators, pus discharge was detected from all of the right tibial pin tracts and the most proximal left tibial pin tracts (Fig. 7). Bacteriological tests detected MRSA. The diagnosis was osteomyelitis, and surgical debridement was performed three times, but the infection in his right tibia persisted. Finally, the infected soft tissue was resected, and a gastrocnemius muscle flap was transplanted into the medullary cavity through a 10×30 mm cortical bone fenestration (Fig. 8). From the time of the first debridement, intravenous daptomycin had been administered for 6 weeks (350 mg/day), followed by oral rifampicin and fosfomycin for 4 months. No recurrence was identified during the 2 years following the last surgery (Fig. 9).

## Discussion

Although the incidence of PTI has been reported to range from 3 % to more than 80 % [3], it rarely evolves into a major infection. It is classified according to the Checketts–Otterburn classification [6]. Grades 1 to 3, classified as minor infections, are the most frequent and usually heal following debridement, antibiotics, and/or pin removal. In contrast, the two cases reported here were classified as grade 6 (major infections), which are relatively rare. That is, the pin tract healed initially after fixator removal but became infected, producing a pus discharge months later. We currently use temporary external fixators for treating the 20 to 30 lower limb fractures that we encounter per year. The incidence of major infections, such as osteomyelitis, has been no more than 1 % during the past 5 years in our institution (unpublished data).

PTI should not be considered a minor complication because once osteomyelitis has developed its treatment entails measures that are more aggressive than curettage of the pin tract. Nevertheless, PTI is a common complication of external fixation. Kazmers *et al*. [7] pointed out that there is no consensus regarding the optimal measures for preventing PTI. In addition, there is a paucity of randomized controlled trials and meta-analyses, as well as a lack of control groups in many studies. Although studies have evaluated a number of variables (that is, cleansing solutions, dressing types, frequency of cleaning), it has been difficult to isolate the effect of any one variable in the event of a positive result.

Parameswaran *et al*. [8] reported a 3.9 % incidence of PTI associated with the use of ring fixators, which differs significantly from the 12.9 % incidence associated with the use of unilateral fixators. This discrepancy suggests that the rate of PTI associated with the use of wires is lower than that for PTI associated with the use of half-pins. In addition, extra attention is needed for patients with poorly controlled glycemia, such as in the patient described in our Case 1. This is true even when using fine wires because definitive ring fixators are usually applied for long periods and diabetes is a risk factor for PTI [9]. Several researchers have promoted the use of pin materials to prevent PTI. Qu *et al*. [10] reported that there were no signs of infection around percutaneous pins coated with a micron-thin sol–gel/triclosan film in distal rabbit tibias. Shirai *et al*. [11] developed techniques to coat titanium implant surfaces with iodine. They concluded that iodine-supported titanium pins could decrease the PTI rate and had no impact on thyroid function. It is expected that these techniques and materials will be applied to patients in the future.

Bibbo and Brueggeman [9] reported that care should be taken to ensure that, during pin insertion, proper pin cooling and, in the case of half-pins, predrilling should be performed to prevent thermal osteonecrosis and skin irritation. Ktistakis *et al*. [12] noted that meticulous surgical technique is essential for preventing infection. For example, they recommended the use of a well-positioned skin incision, a non-touch technique for wire handling, advancement of the pin into the soft tissue by tapping rather than drilling, predrilling, and washing out bone debris before inserting half-pins. Prevention of thermal osteonecrosis is considered a target for improvement in our institution because we do not perform cooling during pin insertion by drilling.

It is recommended that the pin site be treated daily by cleansing the pin–skin interface with simple isopropyl alcohol, freshening the wound edges with sharp dissection, and closing the wound primarily with standard closure techniques after removing the pins [9]. Several reports recommend chlorhexidine as a cleansing agent for pin sites. Cam *et al*. [13] compared a 10 % povidone–iodine solution and chlorhexidine 2 mg/ml and reported that PTI was observed in 27.9 % of the povidone–iodine group and in 9.2 % of the chlorhexidine group. W-Dahl and Toksvig-Larsen [6] compared a chlorhexidine 2 mg/ml solution and sodium chloride 9 mg/ml solution and reported that PTI was found in 9 % of the chlorhexidine group and in 17 % of the sodium chloride group. Polyhexamethylene biguanide or 1 % silver sulfadiazine-impregnated gauze dressing of the pin site has been recommended to prevent PTI [14, 15]. We routinely use once-daily pin site care with chlorhexidine 2 mg/ml solution, but we need to search further to determine whether it is the most appropriate method.

In case 2, primary wound closure was performed successfully using a gastrocnemius muscle flap and skin grafting. The abundant blood flow provided by the muscle flap is an advantage because it delivers antibiotics into the medullary cavity to help control the infection [16]. The patient reported no complaints related to gait or activities of daily living other than mild weakness during ankle planter flexion. The gastrocnemius muscle flap is a useful treatment option for refractory osteomyelitis because the flap harvest causes less functional disturbance and is a relatively easy surgical technique.

## Conclusions

We report two cases of MRSA-induced osteomyelitis of the tibia after PTI. Delayed osteomyelitis with MRSA is uncommon as it is a highly virulent bacteria and leads to early infection. PTI should not be considered a minor complication because once osteomyelitis has developed more aggressive treatment is required as simple curettage is likely to fail. Local pin care is important to prevent infection. Furthermore, we suggest that avoiding thermal osteonecrosis during pin insertion and controlling systemic comorbidities such as diabetes are also important for preventing PTI. The gastrocnemius flap is a useful treatment option for refractory osteomyelitis because flap harvest causes less functional disturbance and is a relatively easy surgical technique.

## Abbreviations

MRSA: Methicillin-resistant *Staphylococcus aureus*
PTI: Pin tract infection
